# QTL and candidate gene analysis unveil genetic control of floret aliphatic glucosinolate side-chain modification in *Brassica oleracea* through multiparent F₂ populations

**DOI:** 10.1093/hr/uhaf232

**Published:** 2025-09-03

**Authors:** Yusen Shen, Mengfei Song, Jiansheng Wang, Xiaoguang Sheng, Huifang Yu, Sifan Du, Shuting Qiao, Honghui Gu

**Affiliations:** Institute of Vegetables, Zhejiang Academy of Agricultural Sciences, Hangzhou 310021, China; Institute of Vegetables, Zhejiang Academy of Agricultural Sciences, Hangzhou 310021, China; Institute of Vegetables, Zhejiang Academy of Agricultural Sciences, Hangzhou 310021, China; Institute of Vegetables, Zhejiang Academy of Agricultural Sciences, Hangzhou 310021, China; Institute of Vegetables, Zhejiang Academy of Agricultural Sciences, Hangzhou 310021, China; Institute of Vegetables, Zhejiang Academy of Agricultural Sciences, Hangzhou 310021, China; College of Life Sciences, China Jiliang University, Hangzhou 310018, China; Institute of Vegetables, Zhejiang Academy of Agricultural Sciences, Hangzhou 310021, China; Sanya Institute, China Agricultural University, Yazhou Bay, Sanya 572025, China; Institute of Vegetables, Zhejiang Academy of Agricultural Sciences, Hangzhou 310021, China

## Abstract

Glucosinolates (GSLs) are sulfur-containing metabolites in *Brassica* species with dual roles in plant defense and human health. While glucoraphanin (GRA) offers anticancer benefits, progoitrin (PRO) poses risks due to goitrogenic effects. This study aimed to dissect the genetic basis of GRA, gluconapin (GNA), and PRO accumulation in florets of *Brassica oleracea* by integrating linkage mapping and quantitative trait locus (QTL) analysis using two F₂ populations (JB-F₂ and GJ-F₂) derived from crosses between broccoli, Chinese kale, and purple cauliflower. High-density linkage maps were constructed using a 10 K SNP array, and GSL profiles were quantified via high-performance liquid chromatography. QTL mapping identified 23 significant loci across both populations, with major-effect QTL clusters on chromosomes C3 and C9. Notably, epistatic analysis revealed strong interactions between major QTLs, particularly between loci on chromosomes C3 and C9, further emphasizing their central role in regulating GSL biosynthesis. Functional analysis prioritized *BolC9t53177H* (homologous to *AOP2*) and *BolC3t13531H* (homologous to *GSL-OH*) as key genes governing GRA-to-GNA and GNA-to-PRO conversions, respectively. Sequence variations in these genes explained parental GSL profiles: A 2-bp deletion causing a frameshift mutation in *BolC9t53177H* disrupted GRA metabolism in broccoli (B58-6), while defective *BolC3t13531H* in Chinese kale (J1402) abolished PRO synthesis. KASP markers developed for these loci enabled efficient genotyping of 104 *B. oleracea* accessions, revealing significant associations with GSL content. This study provides genetic insights and molecular tools to optimize GSL composition, facilitating the breeding of high-GRA/low-PRO *Brassica* varieties with enhanced nutritional value.

## Introduction

Glucosinolates (GSLs), a class of sulfur-containing secondary metabolites in *Brassicaceae* plants, exhibit dual roles in plant defense and human health through their hydrolysis products, such as isothiocyanates and indole derivatives [1]. Sulforaphane, derived from glucoraphanin (GRA), activates the Nrf2 pathway, demonstrating potent anticancer, antioxidant, and anti-inflammatory activities, making it a key compound in functional food and pharmaceutical research [2, 3]. GSLs could also show certain adverse effects. For example, the GSL component progoitrin (PRO) is metabolized to yield goitrin—a sulfur-containing oxazolidine that suppresses thyroid hormone synthesis, ultimately triggering goiter formation while impairing fertility and growth [4]. This double-edged nature of GSLs underscores the urgency of fine-tuning the GRA/PRO ratio via allele-specific editing to optimize the balance between beneficial (e.g. GRA) and harmful (e.g. PRO) components.

GSLs are classified into three major categories based on their precursor amino acids [5, 6]: aliphatic GSLs derived from alanine (Ala), leucine (Leu), isoleucine (Ile), valine (Val), or methionine (Met); aromatic GSLs originating from phenylalanine (Phe) or tyrosine (Tyr); and indolic GSLs synthesized from tryptophan (Trp). Taking aliphatic GSLs as an example, their biosynthesis involves three distinct phases: (i) side chain elongation of specific precursor amino acids, (ii) core structure formation, and (iii) secondary modifications of the amino acid-derived side chains. Extensive structural diversification through side chain elongation and secondary modifications has led to remarkable chemical diversity, with over 130 structurally characterized GSL variants identified across plant species [7].

In the model plant *Arabidopsis thaliana*, genes involved in GSL biosynthesis have been systematically identified, and their metabolic pathways have been well-characterized [5]. For instance, key regulators include *GSL-ELONG* controlling aliphatic GSL side-chain elongation [8], *GSL-OH* mediating PRO synthesis [9], and *AOP2/AOP3* catalyzing the conversion of methylsulfinylalkyl GSLs to alkenyl or hydroxyalkyl derivatives [10]. QTL mapping serves as a cornerstone for unraveling the genetic architecture of complex metabolic traits, particularly in polygenic GSL pathways. Recent studies in *Brassica* species have identified several QTLs associated with GSL metabolism. Key genes such as *MYB28*, *MYB51*, and *GSL-OH* have been pinpointed for their roles in regulating GSL accumulation in seeds and other tissues [11–14]. For example, QTL mapping studies have linked specific genes to seed GSL content, with *BnaC2.MYB28* playing a critical role in regulating seed GSL content in *B. napus* [12]. In addition, metabolic engineering strategies have focused on genes like *AOP2* and *ESP* to boost beneficial GSL metabolites such as GRA and ITCs in Chinese kale. CRISPR/Cas9-mediated editing of *BoaAOP2* significantly enhanced GRA levels [15], while downregulating *BoESP2* increased ITC content by altering the GSL metabolic pathway in Chinese kale sprouts [16]. Despite these advances, most QTL mapping and engineering efforts focus on seeds, sprouts, or leaves. There remains a notable gap in QTL mapping for GSL regulation in floral organs, which limits the application of these findings to curd-type crops like broccoli and cauliflower. Since florets are the primary edible organs of broccoli and cauliflower, they represent the most relevant target for nutritional enhancement in these curd-type crops.

**Figure 1:**
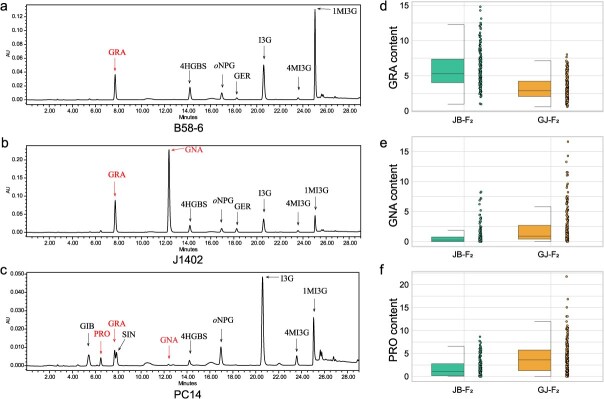
HPLC chromatograms of glucosinolates in parental lines and phenotypic distribution of three major aliphatic glucosinolates in two F_2_ populations. (a–c) HPLC chromatograms of glucosinolates in B58–6, J1402, and PC14. Minor peaks corresponding to low-abundance or functionally irrelevant glucosinolates were not labeled. (d–f) Box plots with scatter points showing GRA, GNA, and PRO content distributions in JB-F_2_ and GJ-F_2_ populations.

*B. oleracea* encompasses a wide range of vegetable crops, including broccoli (*B. oleracea* var. *italica*), cauliflower (*B. oleracea* var. *botrytis*), Chinese kale (*B. oleracea* var. *alboglabra*), and others, which differ markedly in their GSL profiles. Among these, broccoli is renowned for its high GRA content, but it exemplifies a key challenge: the beneficial GRA content varies drastically across genotypes [17], and some cultivars even accumulate excessive amount of the harmful compound PRO [18], thereby limiting their nutritional value. Conventional breeding programs prioritize morphological traits, such as curd color, curd compactness, bud size, and so on. While theoretical frameworks for precisely modulating GSL composition are lacking, hindering progress in nutritional quality improvement. Deciphering the genetic mechanisms underlying GSL metabolism, particularly the synthesis, modification, and dynamic equilibrium of key components, is therefore critical for breeding *Brassica* varieties with enhanced health benefits and minimized risks.

In this study, we constructed two F_2_ populations derived from biparental crosses involving three *B. oleracea* lines: one between Chinese kale and broccoli, and the other between a purple cauliflower landrace and Chinese kale. These two F_2_ populations were used to dissect the genetic regulation of GRA-, GNA-, and PRO-GSL metabolism. Two linkage maps were constructed using a 10 K multiple nucleotide polymorphism (MNP) array, and the GSL contents in florets of the two F_2_ populations and their three parental lines were determined using high-performance liquid chromatography (HPLC) method. Candidate gene mining, gene sequencing amplification, and amino acid variation analysis revealed that *BolC9t53177H* (an *AOP2* homolog) and *BolC3t13531H* (a *GSL-OH* homolog) might be the key genes regulating the transition from GRA to GNA, and subsequently from GNA to PRO in florets of *B. oleracea* species. Furthermore, Kompetitive Allele-Specific PCR (KASP) markers were developed from resequencing data offer practical tools for marker-assisted breeding. By delineating the QTL regulatory network of GSL metabolism in *B. oleracea*, this study not only advances the breeding of high-GRA/low-PRO varieties but also enriches our understanding of metabolic evolution in *Brassicaceae* crops.

## Results

### Determination of the GRA, GNA, and PRO contents and their ratios in two F_2_ populations

The contents of GRA, GNA, and PRO in three parental lines (B58-6, J1402, and PC14) and two F_2_ populations were quantified via HPLC. In B58–6, GRA was exclusively detected, with GNA and PRO below the limit of quantification (Fig. 1a). In contrast, J1402 accumulated both GRA and GNA but lacked detectable PRO (Fig. 1b). Notably, PC14 exhibited all three aliphatic GSLs (GRA, GNA, and PRO) albeit with trace-level GNA (Fig. 1c). Strikingly, although PRO was undetectable in both B58-6 and J1402, phenotypic variation in PRO content was observed in their F_2_ progenies (Fig. 1f and Table 1). This phenomenon may arise because the PRO synthesis pathway requires two or more genes to be fully functional, whereas each parental line (B58-6 and J1402) possesses only one functional gene in the pathway. In the F_2_ generation derived from their hybridization, some individuals retained all functional genes, others lost, and some retained only one. This genetic segregation resulted in extensive phenotypic variation across the population (0–8.65 μmol/g).

**Table 1:** Statistical analysis of phenotypic data for GRA, GNA, PRO, and their ratios in parental lines and two F_2_ populations.

**Populations**	**Trait**	**J1402 (μmol/g)**	**B58-6** ^a^ **(μmol/g)**	**Range (μmol/g)**	**Mean ± S.D. (μmol/g)**	**Skewness**	**Kurtosis**
JB-F_2_	GRA	6.68	2.26	0.96–14.81	5.90 ± 2.64	0.95	0.65
	GNA	13.66	0	0–8.29	0.80 ± 1.50	3.15	10.53
	PRO	0	0	0–8.65	1.78 ± 1.93	1.27	0.98
	GNA/GRA	2.04	0	0–1.95	0.15 ± 0.28	3.55	15.63
	(PRO+GNA)/GRA	2.04	0	0–2.18	0.49 ± 0.44	1.39	2.68
	PRO/GNA	0	-	0–43.69	7.09 ± 7.77	1.84	4.22
	PRO/(GNA + GRA)	0	0	0–1.3	0.31 ± 0.31	1.15	1.04
GJ-F_2_		PC14 (μmol/g)	J1402 (μmol/g)				
	GRA	0.53	6.68	0.6–8.01	3.25 ± 1.62	0.68	−0.25
	GNA	0.03	13.66	0–16.65	2.25 ± 2.99	2.13	4.70
	PRO	0.24	0	0–21.77	4.06 ± 3.41	1.43	3.53
	GNA/GRA	0.06	2.04	0–9.74	0.72 ± 1.03	4.12	27.67
	(PRO+GNA)/GRA	0.51	2.04	0.69–12.55	2.21 ± 1.73	3.36	14.32
	PRO/GNA	8.00	0	0–55.95	6.97 ± 7.67	2.35	9.26
	PRO/(GNA + GRA)	0.43	0	0–6.07	1.06 ± 0.87	1.45	5.17

a The dash in the table indicates that both PRO and GNA were undetected, and therefore the ratio could not be calculated.

The accumulation of GRA, GNA, PRO, and their ratios exhibited extensive variations in both JB-F_2_ and GJ-F_2_ populations, as illustrated in Fig. 1 and Table 1. For core components: GRA in JB-F_2_ showed a broader range (mean 5.90 ± 2.64 μmol/g), while GJ-F_2_ exhibited a more uniform distribution (mean 3.25 ± 1.62 μmol/g). GNA in JB-F_2_ was highly constrained (0–8.29 μmol/g), with most individuals near zero, whereas GJ-F_2_ displayed an extended range (0–16.65 μmol/g) and enhanced variability. PRO was dispersed in both populations, but GJ-F_2_ had a wider range (0–21.77 μmol/g) and a higher proportion of high-PRO phenotypes.

In addition, the ratios among these substances were calculated, including GNA/GRA, (PRO+GNA)/GRA, PRO/GNA, and PRO/(GNA + GRA), in order to explore more genes that might affect the degradation of GRA and the production of PRO. Ratio traits further revealed population-specific patterns: GNA/GRA and (PRO+GNA)/GRA in GJ-F_2_ population exhibited considerable variability, with the box plots showing wide ranges, while PRO/GNA showed pronounced skewness and PRO/(GNA + GRA) approached symmetry, likely governed by polygenic regulation (Supplementary Fig. S1).

Overall, the centralized phenotypic distributions observed in JB-F_2_ may reflect conserved genetic backgrounds, while the heightened phenotypic diversity in GJ-F_2_ suggests potential dominant allele effects or adaptive selection pressures. These patterns provide critical insights into the genetic architecture underlying complex traits.

### High-density linkage maps construction for the two F_2_ populations

The parental lines and F_2_ individuals were genotyped using the customized 10 K MNP array of *B. oleracea*. After several steps of screening, all SNPs could be assigned to nine linkage groups, corresponding to the nine chromosomes of the *B. oleracea*.

**Figure 2:**
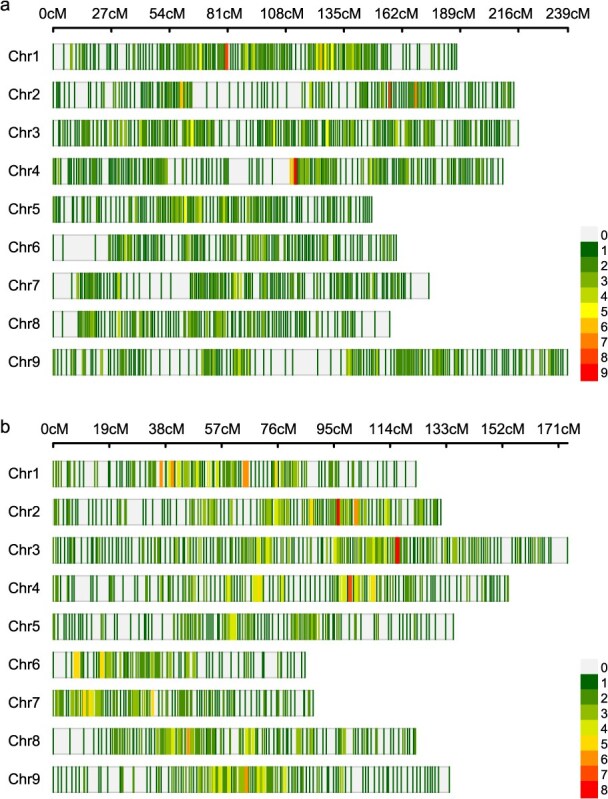
High-density genetic maps constructed using a 10 K MNP array for JB-F_2_ and GJ-F_2_ populations. (a) The distribution of SNP markers on chromosomes in the JB-F_2_ population. (b) The distribution of SNP markers on chromosomes in the GJ-F_2_ population. The color represents the number of markers in 1 cM interval.

In JB-F_2_ population, a total of 1738 filtered SNPs were used to construct a high-density linkage map with a total length of 1702.50 cM. The average distance between adjacent markers was 0.98 cM, corresponding to a physical distance of approximately 0.30 Mb on the reference genome (Fig. 2a). Similarly, for GJ-F_2_ population, a total of 1475 filtered SNPs were used to construct the linkage map, covering a total length of 1147.10 cM. The genetic distance and physical distances between adjacent markers were 0.78 cM and 0.36 Mb, respectively (Fig. 2b). Supplementary Table S1 compiles chromosome-wide genetic marker profiles for JB-F_2_ and GJ-F_2_ populations.

### QTL analysis for GRA, GNA, PRO, and their ratios in two F_2_ populations

QTL mapping was conducted using Windows QTL Cartographer v2.5 [19], and the LOD thresholds were determined through 1000 permutation tests implemented within the software (Supplementary Table S2).

GRA is beneficial to human health, whereas PRO is a harmful component, and gluconapin (GNA) serves as an intermediate in their interconversion. Investigating the genetic regulation of their content ratios is critical for understanding metabolic balance. In this study, we analyzed three basic traits (GRA, GNA, and PRO) and four derived ratios: GNA/GRA and (PRO+GNA)/GRA (representing GRA degradation capacity), as well as PRO/GNA and PRO/(GNA + GRA) (representing PRO synthesis capacity). QTL mapping was performed for these traits in both JB-F_2_ and GJ-F_2_ populations. Notably, 23 significant QTLs (exceeding LOD thresholds) were identified in each population (Table 2).

**Table 2:** Summary of the QTLs identified in JB-F_2_ and GJ-F_2_ populations.

**Populations**	**QTL name** ^a^	**Trait**	**Chr.**	**Pos.**	**LOD**	**Add.**	***R*** ^**2**^	**C.I. (cM)**	**P.I. (Kb)**
**JB-F**_**2**_	qGRA.C2–1	GRA	C2	185.3	7.04	0.89	8.29	184.0–188.3	51 925–59 055
	qGRA.C4–1	GRA	C4	36.1	5.08	0.76	6.02	33.8–37.8	7334–9844
	qGRA.C4–2	GRA	C4	46.1	3.30	0.63	4.00	45.2–47.2	12 201–12 788
	qGRA.C6–1	GRA	C6	105.6	5.47	0.75	6.34	101.6–107.3	35 711–38 137
	qGRA.C7–1	GRA	C7	115.7	4.56	0.71	5.56	114.3–120.7	49 237–50 010
	**qGRA.C9–1**	**GRA**	**C9**	**3.8**	**8.54**	**−1.05**	**11.28**	**0.0–9.1**	**0–1825**
	**qGNA.C3–1**	**GNA**	**C3**	**26.3**	**12.75**	**0.84**	**21.80**	**24.3–27.3**	**3814–4516**
	**qPRO.C3–1**	**PRO**	**C3**	**31.2**	**11.23**	**−0.87**	**14.42**	**28.9–33.2**	**4639–5369**
	qPRO.C7–1	PRO	C7	97.7	3.32	0.45	4.18	96.8–100.4	44 603–45 744
	qPRO.C7–2	PRO	C7	103.8	3.95	0.49	4.94	101.5–110.3	45 805–48 066
	qPRO.C9–1	PRO	C9	8.3	5.93	0.66	8.61	6.0–13.5	1516–2514
	**qG_G.C3–1**	**GNA/GRA**	**C3**	**29.8**	**16.82**	**0.17**	**24.78**	**29.4–33.0**	**4835–5369**
	**qG_G.C9–1**	**GNA/GRA**	**C9**	**3.8**	**8.97**	**0.12**	**13.13**	**2.0–7.2**	**1005–1740**
	qG_G.C9–2	GNA/GRA	C9	15.0	5.83	0.10	8.85	14.3–17.1	2567–3070
	qPG_G.C5–1	(PRO+GNA)/GRA	C5	67.1	3.58	0.09	3.07	66.9–76.4	22 029–33 739
	qPG_G.C5–2	(PRO+GNA)/GRA	C5	79.1	3.58	0.09	3.07	78.4–88.4	33 402–40 238
	**qPG_G.C9–1**	**(PRO + GNA)/GRA**	**C9**	**8.3**	**22.97**	**0.31**	**36.65**	**8.2–9.9**	**1516–1921**
	qP_G.C2–1	PRO/GNA	C2	200.9	3.75	2.25	5.48	200.5–203.9	61 811–62 239
	qP_G.C2–2	PRO/GNA	C2	209.1	4.18	2.26	6.06	208.2–210.4	62 474–62 766
	**qP_G.C3–1**	**PRO/GNA**	**C3**	**29.8**	**7.38**	**−3.91**	**17.63**	**28.0–33.3**	**4516–5369**
	**qP_GG.C3–1**	**PRO/(GNA + GRA)**	**C3**	**29.8**	**14.15**	**−0.15**	**16.10**	**28.9–31.1**	**4639–4961**
	qP_GG.C7–1	PRO/(GNA + GRA)	C7	63.6	4.77	0.08	4.70	54.6–63.7	28 106–29 166
	**qP_GG.C9–1**	**PRO/(GNA + GRA)**	**C9**	**8.3**	**14.40**	**0.16**	**19.26**	**8.2–9.9**	**1516–1921**
**GJ-F**_**2**_	**qGRA.C2–2**	**GRA**	**C2**	**99.7**	**6.67**	**−0.61**	**10.75**	**99.0–100.1**	**48 775–50 025**
	qGRA.C5–1	GRA	C5	63.2	3.95	−0.66	5.56	61.7–64.6	23 245–34 077
	qGRA.C7–2	GRA	C7	49.6	4.64	−0.49	7.05	43.4–54.2	46 263–50 952
	qGNA.C2–1	GNA	C2	14.5	3.31	0.72	3.37	11.3–21.6	2220–3020
	qGNA.C3–2	GNA	C3	21.0	4.45	0.98	4.20	17.4–23.1	3051–3921
	qGNA.C3–3	GNA	C3	30.6	5.37	−1.19	9.57	28.6–34.1	4867–5541
	qPRO.C3–2	PRO	C3	22.1	3.17	0.95	4.99	21.4–24.2	3641–4308
	qPRO.C3–3	PRO	C3	33.1	5.40	1.26	8.31	33.0–36.3	5436–5926
	qPRO.C3–4	PRO	C3	41.0	4.90	1.22	7.58	38.9–43.6	6465–7595
	qPRO.C5–1	PRO	C5	63.2	3.98	−0.85	4.57	62.5–65.3	35 155–34 529
	qPRO.C7–3	PRO	C7	45.1	4.83	−0.91	5.46	41.2–50.5	45 142–48 452
	qPRO.C7–4	PRO	C7	60.3	3.88	−0.80	4.43	57.2–60.6	51 757–53 337
	qPRO.C9–2	PRO	C9	44.0	4.43	0.89	4.94	42.1–47.2	5553–7482
	qPRO.C9–3	PRO	C9	50.1	4.09	0.89	4.57	49.3–52.3	8574–11 192
	qG_G.C3–2	GNA/GRA	C3	30.6	5.70	−0.42	9.66	28.9–39.1	4867–6465
	qG_G.C5–1	GNA/GRA	C5	52.7	3.58	−0.24	4.43	50.3–55.7	7511–9370
	qPG_G.C2–1	(PRO+GNA)/GRA	C2	60.6	4.86	0.47	5.73	58.9–66.3	12 385–14 776
	**qPG_G.C5–3**	**(PRO + GNA)/GRA**	**C5**	**52.0**	**6.77**	**−0.63**	**10.53**	**51.6–53.4**	**7926–8922**
	**qPG_G.C9–2**	**(PRO + GNA)/GRA**	**C9**	**46.8**	**9.42**	**0.66**	**10.71**	**43.2–48.9**	**5980–7708**
	**qP_G.C3–2**	**PRO/GNA**	**C3**	**31.6**	**27.89**	**6.61**	**40.91**	**30.4–36.2**	**4961–5926**
	**qP_GG.C3–2**	**PRO/(GNA + GRA)**	**C3**	**30.6**	**9.39**	**0.46**	**14.61**	**30.1–33.0**	**4961–5436**
	**qP_GG.C3–3**	**PRO/(GNA + GRA)**	**C3**	**35.9**	**10.19**	**0.45**	**15.72**	**35.0–36.6**	**5840–6359**
	**qP_GG.C3–4**	**PRO/(GNA + GRA)**	**C3**	**41.4**	**9.24**	**0.44**	**14.39**	**38.4–42.0**	**6359–7009**

a Major-effect QTLs (bold) account for >10% phenotypic variation. Trait abbreviations: G_G = GNA/GRA; PG_G = (PRO+GNA)/GRA; P_G = PRO/GNA; P_GG = PRO/(GNA + GRA).

**Figure 3:**
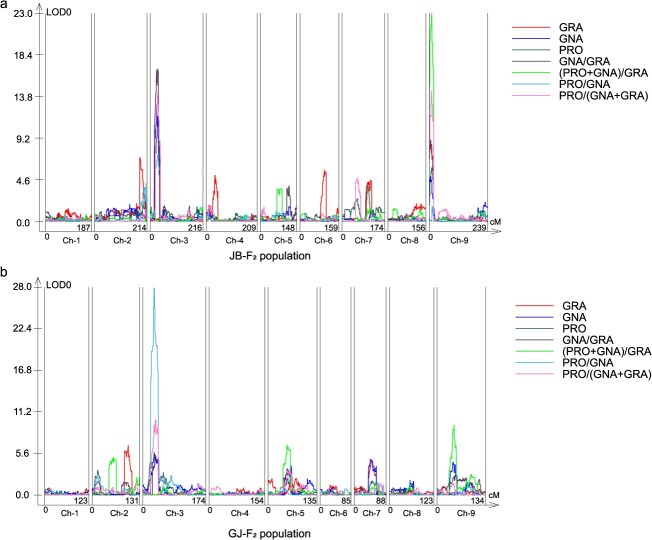
QTL mapping results of glucosinolate components (GRA, GNA, PRO) and their ratios in two F_2_ populations. (a) QTL localization for glucosinolate components and ratios in the JB-F_2_ population. (b) QTL localization for glucosinolate components and ratios in the GJ-F_2_ population.

In the JB-F_2_ population, nine major-effect QTLs (*R*^2^ > 10%) were detected, primarily clustered on chromosomes C3 and C9 (Fig. 3a). Chromosome C3 exhibited a prominent QTL enrichment, harboring five high-impact loci: *qGNA.C3–1* (GNA, *R*^2^ = 21.80%), *qPRO.C3–1* (PRO, *R*^2^ = 14.42%), *qG_G.C3–1* (GNA/GRA, *R*^2^ = 24.78%), *qP_G.C3–1* (PRO/GNA, *R*^2^ = 17.63%), and *qP_GG.C3–1* (PRO/(GNA + GRA), *R*^2^ = 16.10%). These QTLs displayed a mean LOD of 12.47, with *qG_G.C3–1* achieving the highest LOD (16.82), underscoring their statistical robustness. Their confidence intervals (24.3–33.3 cM) spanned a 3.8–5.4 Mb physical region on C3, suggesting a pivotal genetic hub regulating the three components. On C9, three of four major QTLs were ratio-related, with *qPG_G.C9–1* ((PRO+GNA)/GRA, *R*^2^ = 36.65%, LOD = 22.97) showing the strongest effect. Its narrow interval (8.2–9.9 cM; 1.5–1.9 Mb) implies a potential regulatory hotspot for multitrait coordination (Table 2).

The GJ-F_2_ population harbored seven major QTLs on chromosomes C2, C3, C5, and C9 (Fig. 3b). The most impactful locus, *qP_G.C3–2* (PRO/GNA, *R*^2^ = 40.91%, LOD = 27.89), exhibited a strong additive effect (6.61) and spanned 30.4–36.2 cM (4.9–5.9 Mb), indicating a key regulatory element. In contrast, *qPG_G.C9–2* ((PRO+GNA)/GRA, *R*^2^ = 10.71%, LOD = 9.42) on C9 showed lower contribution and lacked replication across populations (Table 2).

Comparative analysis revealed partially overlapping QTL clusters on chromosome C3 in both F₂ populations, specifically spanning 3.8–5.4 Mb in JB-F₂ and 4.9–5.9 Mb in GJ-F₂, suggesting a conserved but shifted regulatory region between populations. In JB-F₂, this region harbors five QTLs (*qGNA.C3–1*, *qPRO.C3–1*, *qG_G.C3–1*, *qP_G.C3–1*, *qP_GG.C3–1*) associated with GNA and PRO content, explaining on average 18.95% of the phenotypic variation (mean LOD = 12.47). Similarly, in GJ-F₂, four QTLs (*qGNA.C3–3*, *qPRO.C3–3*, *qP_G.C3–2*, *qP_GG.C3–2*) were identified in the same region, with an average phenotypic contribution of 18.35% and mean LOD of 12.01 (Table 2). The consistent detection of QTLs for similar traits across both populations indicates a conserved regulatory locus that likely modulates the metabolic conversion of GNA to PRO.

In contrast, QTL clusters on chromosome C9 were located in distinct physical regions. In JB-F₂, three QTLs (*qG_G.C9–1*, *qPG_G.C9–1*, *qP_GG.C9–1*) associated with GRA, GNA, or related ratios were located between 0 and 1.9 Mb, accounting for an average phenotypic variation of 23.01% (mean LOD = 15.45). In GJ-F₂, two QTLs identified between 5.9–7.7 Mb explained a lower average phenotypic variation of 7.82% (mean LOD = 6.92) (Table 2). This divergence highlights the population specificity of C9 QTLs and suggests genetic background–dependent variation in locus expression and localization.

### Detection of epistatic interactions between QTLs

A total of nine and six pairs of epistatic QTLs were identified in the JB-F_2_ and GJ-F_2_ populations, respectively (Table 3). Among these interactions, 12 out of the 15 epistatic pairs (80%) involved at least one major-effect QTL, suggesting that epistasis often occurs in conjunction with primary loci. In the JB-F_2_ population, three epistatic interactions showed extremely strong effects (EPI_JB_3, EPI_JB_4, and EPI_JB_9), all with *P*-values lower than 1.00E-07. Notably, all QTLs involved in these strong interactions were located in QTL clusters on chromosomes C3 and C9, highlighting the strong epistatic potential between these two chromosomal regions. In the GJ-F_2_ population, a significant QTL interaction was detected for the trait (PRO+GNA)/GRA. The strongest interaction was observed between qPG_G.C5-3 and qPG_G.C9-2 (*P* = 3.52E-06). These QTLs were located on chromosomes C5 and C9, respectively, suggesting a potential regulatory interaction between these loci in modulating GRA degradation pathways in *Brassica* vegetables.

**Table 3:** Epistatic interactions were detected between QTLs.

**Populations**	**Epistatic interactions**	**Trait**	**QTL1** ^**a**^	**QTL2** ^**b**^	***P*-value** ^**c**^
JB-F_2_	EPI_JB_1	GRA	qGRA.C7–1	**qGRA.C9–1**	3.43E-02
	EPI_JB_2	PRO	**qPRO.C3–1**	qPRO.C9–1	1.83E-03
	EPI_JB_3	GNA/GRA	**qG_G.C3–1**	**qG_G.C9–1**	**9.08E-19**
	EPI_JB_4	GNA/GRA	**qG_G.C3–1**	qG_G.C9–2	**1.09E-13**
	EPI_JB_5	(PRO+GNA)/GRA	qPG_G.C5–1	**qPG_G.C9–1**	5.05E-03
	EPI_JB_6	(PRO+GNA)/GRA	qPG_G.C5–2	**qPG_G.C9–1**	3.33E-03
	EPI_JB_7	PRO/GNA	qP_G.C2–1	**qP_G.C3–1**	1.28E-03
	EPI_JB_8	PRO/GNA	qP_G.C2–2	**qP_G.C3–1**	1.07E-02
	EPI_JB_9	PRO/(GNA + GRA)	**qP_GG.C3–1**	**qP_GG.C9–1**	**6.00E-08**
GJ-F_2_	EPI_GJ_1	GNA	qGNA.C2–1	qGNA.C3–2	3.86E-02
	EPI_GJ_2	GNA	qGNA.C2–1	qGNA.C3–3	3.48E-02
	EPI_GJ_3	GNA/GRA	qG_G.C3–2	qG_G.C3–2	4.30E-02
	EPI_GJ_4	(PRO+GNA)/GRA	qPG_G.C2–1	**qPG_G.C5–3**	2.47E-05
	EPI_GJ_5	(PRO+GNA)/GRA	qPG_G.C2–1	**qPG_G.C9–2**	2.65E-05
	EPI_GJ_6	(PRO+GNA)/GRA	**qPG_G.C5–3**	**qPG_G.C9–2**	**3.52E-06**

a bold text indicates the major-effect QTLs (PEV > 10%)

b bold text indicates the major-effect QTLs (PEV > 10%)

c bold text indicates the most significant epistatic interactions (*P* < 1.00E-05)

### Candidate genes mining for GRA, GNA, and PRO contents

Candidate genes were mined by integrating homology-based functional annotations from the *A. thaliana* genome with the physical positions of SNPs or genes mapped to the *B. oleracea* HDEM reference genome. Within 46 QTL intervals associated with GSL traits, we identified 5854 annotated genes, among which 31 exhibited functional homology to previously characterized GSL biosynthesis genes (Supplementary Table S3).

To further prioritize candidate regions, we focused on the major overlapping QTL intervals on chromosomes C3 (3.8–5.4 Mb in JB-F₂ and 4.9–5.9 Mb in GJ-F₂) and C9 (0–1.9 Mb in JB-F₂ and 5.9–7.7 Mb in GJ-F₂). In total, 380 genes on C3 and 481 genes on C9 were identified in these intervals (Supplementary Table S4). Among them, only *BolC3t13531H*, orthologous to *Arabidopsis GSL-OH* (*AT2G25450*), was found within QTL intervals on C3 and functionally annotated as a 2-oxoacid-dependent dioxygenase involved in the biosynthesis of 2-hydroxybut-3-enyl GSL, a precursor of PRO [9]. It was recurrently mapped in seven distinct QTLs (*qP_G.C3-1*, *qPRO.C3-1*, *qG_G.C3-1*, *qGNA.C3-3*, *qG_G.C3-2*, *qP_G.C3-2*, and *qP_GG.C3-2*) (Supplementary Table S3), suggesting its potential involvement in PRO-related GSL variation. On C9, a tandem cluster of three *AOP* homologs—*BolC9t53177H*, *BolC9t53178H*, and *BolC9t53179H*—spanning 1 610 291 to 1 619 410 bp, co-occurred in five overlapping QTLs (*qGRA.C9-1*, *qG_G.C9-1*, *qPRO.C-1*, *qPG_G.C9-1*, *qP_GG.C9-1*). Based on sequence similarity, *BolC9t53177H* corresponds to *AOP2* (*AT4G03060*), while *BolC9t53178H* and *BolC9t53179H* align with *AOP1* (*AT4G03070*) (Supplementary Fig. S2). All three genes encode 2-oxoglutarate-dependent dioxygenases, which in *Arabidopsis* are known to function in GSL side-chain modification [10]. Although their specific contributions in *B. oleracea* remain to be elucidated, these genes were prioritized as candidate regulators of GRA, GNA, and PRO diversity.

**Figure 4:**
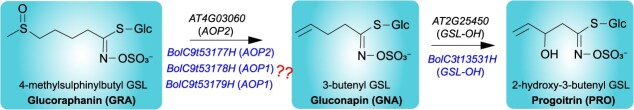
Schematic representation of the biosynthetic pathways for GRA, GNA, and PRO in *A. thaliana* and *Brassica* species. Boxes contain structural formulas and metabolite names of the three compounds. Arrows connecting the metabolites are labeled with regulatory genes from *A. thaliana* (above the arrows) and candidate genes identified in this study (below the arrows).

Taken together, these observations suggest that GSL biosynthetic pathways may be broadly conserved between *B. oleracea* and *A. thaliana*, yet may have diverged functionally among homologous enzyme-encoding genes. To illustrate this hypothesis, we constructed a schematic model (Fig. 4) summarizing the putative enzymatic conversions involved in GRA, GNA, and PRO metabolism, with *Arabidopsis* genes labeled above and *B. oleracea* candidate genes labeled below the arrows.

### Variation analysis of important candidate genes in parental lines

**Figure 5:**
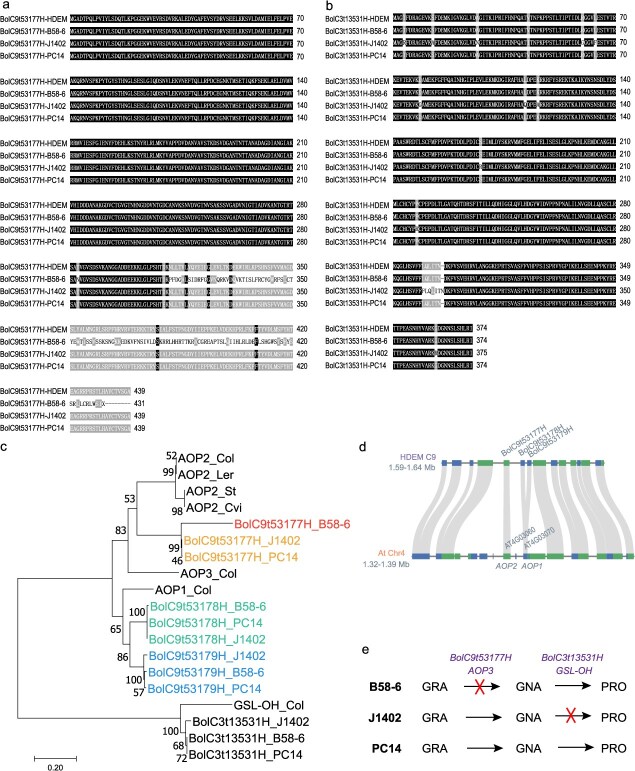
Amino acid variation analysis of candidate genes regulating GRA-to-GNA and GNA-to-PRO conversion and their phenotypic impact in parental lines. (a) Amino acid sequence alignment of *BolC9t53177H* (candidate gene for GRA-to-GNA conversion) in three parental lines (B58–6, J1402, PC14) against the HDEM reference genome. (b) Amino acid sequence alignment of *BolC3t13531H* (candidate gene for GNA-to-PRO conversion) in the same parental lines. (c) Phylogenetic tree analysis comparing *BolC9t53177H*, *BolC9t53178H*, and *BolC9t53179H* in parental lines with *Arabidopsis* homologs *AOP1*, *AOP2*, and *AOP3*. (d) Synteny analysis between the *B*. *oleracea* HDEM genome and *A. thaliana* chromosome 4 reveals the collinearity of *AOP* gene homologs. (e) Schematic diagram illustrating the phenotypic variation patterns influenced by these two candidate genes across the parental lines. The symbol ‘×’ represents that the sequence variation of this gene leads to functional obstruction.

To investigate which of the three tandem *AOP* candidate gene plays a pivotal role in the conversion of GRA to GNA, we amplified DNA sequences of these genes from the parental lines B58-6, J1402, and PC14 using PCR. Comparative analysis with the HDEM reference genome enabled the identification of coding sequences (CDS), which were subsequently translated into amino acid sequences (Supplementary Table S5). Notably, the *BolC9t53177H* gene in line B58-6 exhibited a frameshift mutation caused by a deletion of two A/T nucleotides at positions 1 610 954 and 1 610 955 on chromosome C9 (Fig. 5a). This mutation resulted in aberrant amino acid translation downstream of the deletion site. In contrast, *BolC9t53178H* and *BolC9t53179H* only exhibited synonymous or conservative amino acid changes (Supplementary Fig. S3), suggesting that they are unlikely to cause functional disruption.

To investigate the phylogenetic relationships between the three *AOP* paralogs in *B. oleracea* and *Arabidopsis AOP* genes, we constructed a phylogenetic tree based on amino acid sequences of the three *AOP* genes from three parental lines and their *Arabidopsis* homologs (Fig. 5c). Importantly, given that the *Arabidopsis* Col-0 reference genome carries a non-functional *AOP2* allele, which is absent or severely truncated in some protein databases, we included *AOP2* sequences from four ecotypes (Col, Ler, St, and Cvi), representing both functional and nonfunctional variants [20]. The analysis revealed that *BolC9t53178H* and *BolC9t53179H* in *B. oleracea* clustered more closely with *AOP1* in *Arabidopsis*, consistent with their amino acid sequence alignments (Supplementary Fig. S2). In contrast, *BolC9t53177H* exhibited closer phylogenetic affinity to *Arabidopsis AOP2*, followed by *AOP3* (Fig. 5c)*,* which was also consistent with its amino acid sequence alignments (Supplementary Fig. S2), confirming its assignment as an *AOP2* homolog. Despite this overall conservation, *BolC9t53177H* in B58-6 exhibited weaker clustering with its homologs in J1402 and PC14, potentially reflecting functional variation among parental lines (Fig. 5c).

To further confirm the evolutionary relationship of candidate genes within the C9 QTL region, we performed a synteny analysis between the *B. oleracea* HDEM genome and *A. thaliana* chromosome 4. The genomic region encompassing *BolC9t53177H*, *BolC9t53178H*, and *BolC9t53179H* on HDEM chromosome C9 exhibited conserved collinearity with the *Arabidopsis AOP* locus. Specifically, *BolC9t53177H* aligned with *AOP2*, while *BolC9t53178H* and *BolC9t53179H* showed syntenic correspondence with *AOP1* (Fig. 5d).

Collectively, these findings highlight *BolC9t53177H*, homologous to *A. thaliana AOP2*, as the critical gene governing the GRA to GNA transition among the three *AOP* homologous genes in *B. oleracea*. Notably, while both species exhibit nonfunctional *AOP2* alleles, the underlying mutations differ: *B. oleracea* B58-6 harbors a 2-bp deletion causing a frameshift, whereas *A. thaliana* Col-0 contains larger internal deletions [20]. This suggests conserved functional roles with lineage-specific loss-of-function mechanisms.

Similarly, DNA sequencing of the candidate gene *BolC3t13531H* (*GSL*-*OH*) revealed amino acid variations across the three parental lines (Fig. 5b). The *BolC3t13531H* sequence was highly conserved in B58-6 and PC14, aligning perfectly with the HDEM reference genome. In contrast, J1402 exhibited 13 amino acid alterations, including an insertion of phenylalanine between residues 289 and 290 (Fig. 5b). Consistent with these findings, phylogenetic analysis showed that all three parental alleles clustered with *Arabidopsis* GSL-OH (*AT2G25450*), but the J1402 allele displayed slightly weaker clustering relative to those from B58-6 and PC14, likely due to the observed sequence divergence (Fig. 5c). We hypothesize that these cumulative mutations likely impair the enzymatic function of *BolC3t13531H* in J1402, thereby blocking the conversion of GNA to PRO and resulting in substantial GNA accumulation alongside negligible PRO levels.

Integrating sequence variations of the four candidate genes with the GSL profiles (GRA, GNA, PRO) across the parental lines, we propose the following mechanistic model (Fig. 5e): In B58-6, a 2-bp deletion in *BolC9t53177H* causes a frameshift mutation that disrupts the GRA-to-GNA conversion, leading to GRA accumulation and absence of both GNA and PRO (Fig. 1a). J1402 retains functional *BolC9t53177H* but harbors a defective *BolC3t13531H*, permitting partial GRA-to-GNA conversion while abolishing PRO synthesis (Fig. 1b). PC14, with intact *BolC9t53177H* and *BolC3t13531H*, maintains functional pathways for all three GSLs (Fig. 1c). These results establish *BolC9t53177H* as the key regulator of GRA metabolism and underscore the hierarchical roles of *AOP* homologous genes in GSL biosynthesis.

### KASP markers development for the GRA, GNA, and PRO glucosinolates in *B. oleracea*

**Figure 6:**
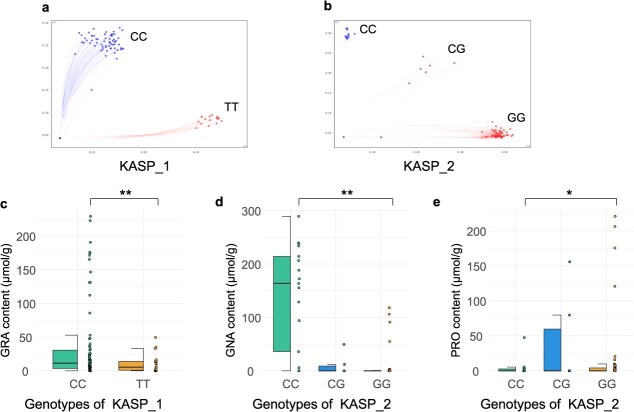
KASP marker-based genotyping and phenotypic variation analysis in *B. oleracea*. (a) Genotyping results of 104 *B. oleracea.* Accessions using the KASP_1 marker developed for the candidate gene *BolC9t53177H*. (b) Genotyping results of the same accessions using the KASP_2 marker developed for the candidate gene *BolC3t13531H*. (c) Phenotypic comparison of GRA content between CC and TT genotypes (KASP_1, ^**^P < 0.01). (d) Phenotypic comparison of GNA content between CC and GG genotypes (KASP_2, ^**^P < 0.01). (e) Phenotypic comparison of PRO content between CC and GG genotypes (KASP_2, ^*^P < 0.05).

Based on candidate gene analysis and locus variation assessment, we developed a KASP marker (designated KASP_1) within the *BolC9t53177H* gene. This marker comprises forward primers (FAM: 5′-GAAGGTGACCAAGTTCATGCTCACACATTTATGCTCCAGAGACGGT-3′; HEX: 5′-GAAGGTCGGAGTCAACGGATTCACACATTTATGCTCCAGAGACGGC-3′) and a reverse primer (Common: 5′-ACTTACGTTGACTTGATGAGTTTC-3′), with universal adaptor sequences added to the 5′ ends of the forward primers. Genotyping of 104 *B. oleracea* accessions (the majority of which were broccoli lines) using this marker revealed two distinct genotypes: TT (23 accessions) and CC (81 accessions) (Fig. 6a). Significant differences (*P* < 0.01) in GRA glucosinolate content were observed between these two genotype groups (Fig. 6c).

Additionally, we designed another KASP marker (KASP_2) targeting the *BolC3t13531H* gene, with the following primer sequences: FAM: 5′-GAAGGTGACCAAGTTCATGCTCCAACCACTATGTGGCTAGAAAAG-3′; HEX: 5′-GAAGGTCGGAGTCAACGGATTCCAACCACTATGTGGCTAGAAAAC-3′; Common: 5′-ACCACTATGTGTGGCTAGAAAAC-3′. Genotyping results showed three genotypes among the 104 *B. oleracea* accessions: CC (17 accessions), CG (6 accessions), and GG (81 accessions) (Fig. 6b). Statistical analysis demonstrated highly significant differences (*P* < 0.01) in GNA glucosinolate content between CC and GG groups (Fig. 6d), while PRO glucosinolate content exhibited significant differences (*P* < 0.05) between two groups (Fig. 6e).

## Discussion

Despite the dual roles of GSLs in plant defense and human health, systematic genetic studies targeting GSL composition in *B. oleracea*—particularly in floral organs of broccoli and related cultivars—remain limited. While beneficial components like GRA have attracted significant attention, their genetic regulation in broccoli is poorly characterized compared to model species like *A. thaliana* [5]. Although key biosynthetic genes, such as *AOPs* and *GSL-OH* have been identified in *Arabidopsis*, their homologs in *B. oleracea* are understudied. Moreover, conventional breeding programs for broccoli prioritize morphological traits (e.g. curd color, curd compactness, bud size), often neglecting metabolic quality traits such as GRA/PRO balance. Even when GSL-related loci are reported [21–23], practical tools like molecular markers for marker-assisted selection remain scarce, hindering the development of nutrient-enhanced varieties. This study addresses these gaps by constructing high-density linkage maps, mapping QTLs for GRA, GNA, and PRO accumulation, and developing KASP markers to bridge genetic insights with breeding applications.

While *F*₂ populations are widely utilized for QTL mapping, their transient nature restricts repeated phenotyping, potentially compromising data reproducibility [24, 25]. To address these limitations, we developed a dual-population strategy by crossing a common parental line (Chinese kale, J1402) with two distinct partners: a broccoli line (B58-6) and a purple cauliflower landrace (PC14). Both F₂ populations were genotyped using a 10 K *Brassica* liquid-phase MNP array, yielding high-density genetic maps with marker densities of 0.98 and 0.78 cM, respectively. This approach builds upon our prior successes in mapping key agronomic traits in *B. oleracea*, including self-incompatibility [24] and curd setting height [26]. Although we have made efforts in noise control within single environment to compensate for the limitations of long-term phenotypic validation in the F_2_ population, in order to consistently identify QTLs across multiple environments and uncover more environment-interacting factors, we are currently developing recombinant inbred line (RIL) populations derived from the same parental crosses (J1402 × B58-6 and PC14 × J1402). These stable RIL populations will be invaluable for confirming the QTL effects observed in this study, fine-mapping the causal genes, and assessing GSL stability. Additionally, given the dynamic interconversion among the three GSL components (GRA, GNA, and PRO), we proposed an innovative approach to map key QTLs regulating their interconversion by utilizing ratio-based metrics. These ratios were designed based on the known sequential transformation of GRA → GNA → PRO in the aliphatic GSL pathway. A higher GNA/GRA or (PRO+GNA)/GRA ratio indicates a greater extent of GRA turnover, while higher PRO/GNA or PRO/(GNA + GRA) values reflect a shift toward PRO biosynthesis. Similar ratio traits have been used in metabolic studies to capture flux trends between precursor and product molecules [15, 27]. In our study, six out of nine (66.7%) major QTLs in the JB-F₂ population and six of seven (85.7%) major QTLs in the GJ-F₂ population were ratio-associated QTLs (Table 2). The reproducibility of co-localized QTLs across independent populations and traits underscores the methodological reliability.

The identification of major QTL clusters on chromosomes C3 and C9 highlights conserved and lineage-specific genetic mechanisms governing GSL metabolism. Notably, strong epistatic interactions were detected among QTLs on these chromosomes, particularly in the JB-F_2_ population, suggesting the existence of interaction hotspots that may coordinate the regulation of complex traits. The overlapping QTL intervals on C3 (3.8–5.9 Mb) across both F_2_ populations suggest a critical genomic hub regulating the metabolic balance between beneficial (GRA) and detrimental (PRO) GSLs. This region harbors *BolC3t13531H*, a homolog of *Arabidopsis GSL-OH*, which catalyzes PRO precursor synthesis. Its conservation across divergent populations underscores its central role in GSL side-chain modification, consistent with findings in *Arabidopsis* [9]. In contrast, population-specific QTLs on C9 reflect genetic background-dependent divergence in *AOP* homologs (*BolC9t53177H*–*BolC9t53179H*). Among the three candidates, *BolC9t53177H* was prioritized as the most likely gene governing the GRA-to-GNA conversion in *Brassica* species, based on an integrated analysis of DNA sequence amplification, amino acid variation, phylogenetic relationships, and synteny conservation.

In *Arabidopsis*, *AOP2* and *AOP3* catalyze the conversion of GRA to GNA, while *AOP1* is functionally ambiguous [10]. To avoid misclassification due to the absence of functional *AOP2* in the Col-0 reference genome, we incorporated *AOP2* alleles from multiple ecotypes with both functional and nonfunctional variants [20]. This improved phylogenetic resolution and confirmed that *BolC9t53177H* is orthologous to *AOP2*, not *AOP3*, as supported by both phylogenetic (Fig. 5c) and synteny analyses (Fig. 5d). Notably, while both *Arabidopsis* and *B. oleracea* show natural *AOP2* loss-of-function mutations, the underlying mechanisms differ: *Arabidopsis AOP2* dysfunction often results from exon deletions or rearrangements, whereas in broccoli, a simple 2-bp deletion causes a frameshift. These observations support a model of conserved enzymatic function with species-specific mutational trajectories. In contrast, *BolC9t53178H* and *BolC9t53179H* align more closely with *AOP1*, as supported by prior BAC-based genome studies in broccoli and collard [28], confirming the presence of *AOP1* in the *B. oleracea* C genome.

Our integrated QTL mapping and candidate gene analysis not only elucidated the differential accumulation of GRA, GNA, and PRO among the three parental lines but also provided mechanistic insights into the unexpected PRO polymorphism observed in the JB-*F*₂ population derived from J1402 and B58-6, both of which lack detectable PRO (Fig. 1a, b, and  f). This phenomenon implies that allelic complementation between the parental lines—broccoli (B58-6) and Chinese kale (J1402)—may inadvertently restore biosynthetic pathways for PRO in hybrid offspring, posing potential risks for undesirable metabolite accumulation during breeding of popular hybrids such as broccolini (a hybrid of broccoli and Chinese kale). To address this challenge, we developed two simple and reliable KASP markers (KASP_1 and KASP_2) within the key candidate genes *AOP2* and *GSL-OH*, respectively. Compared to traditional SSR and InDel markers, KASP genotyping offers higher accuracy (error rate 0.7%–1.6%), lower cost (up to 46% cheaper), and greater flexibility. Its uniplex SNP-based format is well suited for moderate-throughput applications and marker-assisted selection in diverse *Brassica* populations [29, 30]. In this study, the developed KASP markers allow breeders to screen young plants for genetic profiles associated with high GRA and low PRO content early in the breeding process. This approach not only reduces the time and cost of traditional field trials but also ensures the selection of nutritionally superior varieties.

While functional validation of *BolC9t53177H* and *BolC3t13531H* through transgenic complementation or CRISPR-based approaches was not conducted in this study, their roles in GSL metabolism were strongly supported by homology to well-characterized *Arabidopsis* genes (*AOP2*/*AOP3* and *GSL-OH*). Given the high evolutionary conservation of GSL pathways between *Brassica* and *Arabidopsis*, coupled with the significant associations of KASP markers developed here with GSL content, our findings provide robust indirect evidence for their functional relevance in *B. oleracea*. These markers offer immediate utility for marker-assisted breeding to optimize GRA/PRO ratios. Nevertheless, future studies incorporating transgenic or gene-editing techniques could further confirm their precise biochemical roles. Such efforts would deepen our understanding of species-specific regulatory mechanisms in GSL metabolism. In addition, to better understand how GSL-related genes work together, future studies could combine QTL mapping with transcriptomics, proteomics, or metabolomics. These approaches can help identify when and where candidate genes are active, and how they affect metabolite levels. For example, Hirai *et al.* used transcriptome and metabolite data to discover two transcription factors (*MYB28* and *MYB29*) that control GSL production in *Arabidopsis* [31]. Sawada *et al.* also combined gene expression and metabolite analysis to confirm the roles of enzymes involved in side-chain elongation of methionine-derived GSLs [32]. Similar strategies in *B. oleracea* could help confirm the functions of *AOP2* and *GSL-OH*, and find other genes that regulate GSL balance. This would provide a more complete picture of the GSL pathway and support more efficient breeding.

## Materials and methods

### Plant material and growth conditions

Three *B. oleracea* morphotypes, Chinese kale J1402, broccoli B58-6, and a purple cauliflower landrace called PC14 were used as parents to construct two F*_2_* populations. JB-F_2_ population containing 214 lines was developed from a cross between J1402 and B58-6, and GJ-F_2_ population containing 226 lines was developed from a cross between PC14 and J1402.

All plants, including the parental lines (B58-6, J1402, PC14) and both F_2_ populations (JB-F_2_ and GJ-F_2_), were grown in a greenhouse at the Yangdu Experimental Farm of the Zhejiang Academy of Agricultural Sciences (Hangzhou, China) during a single season. Specifically, they were sown in August 2021 and floret samples were harvested in November–December 2021 for GSL analysis. The light, temperature, and humidity in the greenhouse were controlled by external environmental conditions and were not artificially adjusted. The field was managed under conventional agronomic practices, and disease and pest management followed standard field practices to ensure uniform plant development.

### Sample preparation for extracting the glucosinolates

During the reproductive maturation phase (November–December 2021), four spatially stratified secondary inflorescences (~5 cm diameter) were sampled from sides and center of the head of each individual plant. These inflorescences were then wrapped with tin foil and were immediately submerged in liquid nitrogen for rapid freezing. Subsequently, the frozen inflorescences were transferred to a Scientz-100F vacuum freeze-drier where they undergo a freeze-drying process until the samples were dried. Then the dried florets were grounded into a fine powder using a mixer mill (MM400, Retsch) at 30 Hz for 1.5 min, and placed into 50-ml centrifuge tubes, each tube’s bottom lined with desiccant to absorb any residual moisture. The tubes were stored in refrigerator maintained at −20°C, awaiting the extraction of glucosinolates.

### Glucosinolates extraction and quantification

Each biological replicate consisted of a pooled floret sample from an individual plant. From each pooled sample, two independent technical replicates (~200 mg of powder each) were processed through extraction and HPLC analysis. To correct for variation in extraction efficiency and injection volume, 3 mM ortho-nitrophenyl-β-D-galactopyranoside (oNPG, Sigma, St Louis, MO, USA) was used as an internal standard. To ensure consistency and peak identification accuracy, a quality control sample (*A. thaliana* Col-0) was run every 24 injections. The extraction procedure for GSLs was conducted based on our previously published method, with slight adjustments [18, 33]. Specifically: About 200 mg of samples were subjected to boiling for 10 minutes to facilitate the extraction of GSLs. Subsequently, the aqueous extract obtained was loaded onto a DEAE-Sephadex A-25 column. Following equilibration, the column was washed three times using 20 mM pyridine–acetate solution and twice with distilled water. Thereafter, 100 μl of sulfatase (0.1%, equivalent to 1.4 U) was introduced into the column and incubated for a duration of 16 hours or overnight, allowing for the conversion of GSLs into their desulfo analogs. Finally, the desulfo-GSLs were eluted with 1.5 ml of water.

HPLC analysis was carried out using an HPLC system comprising a Waters 600 chromatograph (Waters, USA), equipped with an auto-injector and an UV-visible diode array detector. The composition of GSLs was determined by their retention times, while their concentrations were calculated based on the HPLC peak areas, utilizing published UV response factors for individual desulfo-GSLs at 226 nm [34]. The oNPG peak also served as a retention time reference, and the relative retention time between oNPG and each target compound was used to assist in peak identification across batches. Additionally, oNPG was consistently used as an internal reference to normalize peak area values and ensure comparability among samples.

### Linkage maps construction and QTL mapping

The genomic DNA of individuals from the two F_2_ populations (JB-F_2_ and GJ-F_2_) was extracted from young leaf tissues using a modified CTAB method [35]. A 10 K MNP array containing 72 791 SNPs (unpublished) for *B. oleracea* was used to genotype the JB-F_2_ and GJ-F_2_ populations. We refined the genetic markers by eliminating those that exhibited: (i) no polymorphism between parents, (ii) heterozygosity or missing data in parents, (iii) missing loci in over 10% of the F_2_ offsprings, and (iv) severe segregation distortion. Subsequently, the SNPs were binned using a custom Perl script we developed, and the genetic linkage map was generated using the MSTmap software [36]. Distribution of SNP markers on chromosomes was mapped using CMplot (v4.3.0) package in R language.

QTL mapping analysis was carried out using Windows QTL Cartographer v2.5 with the composite interval mapping (CIM) method [19], and the likelihood of odd (LOD) threshold for determining QTL for GSL was established using 1000 permutations at *P* = 0.05.

### Detection of epistatic interactions between QTLs

Epistatic interactions among QTLs were analyzed using custom R scripts. Genotypic and phenotypic data were processed in R (v4.4.3) using base functions and the packages *dplyr*, *tidyr*, and *readxl*. For each trait, pairwise interaction tests were conducted between QTL regions using a linear model framework. Specifically, two-way ANOVAs were performed for all pairs of QTLs within the same trait, using genotype combinations at the flanking markers as predictors and the phenotypic trait value as the response variable. The significance of interaction effects was assessed using the *P*-value from the interaction term in the model. Results were filtered using a significance threshold (*P* < 0.05), and highly significant interactions (*P* < 1e−5) were highlighted as strong epistatic effects. All analyses were performed separately for each population to identify population-specific epistatic interactions.

### Candidate genes mining for the content of GRA, GNA, and PRO glucosinolates

Candidate genes were predicted through an integrated approach combining QTL mapping and functional annotation. First, flanking markers of each QTL interval were aligned to the *B. oleracea* HDEM reference genome to delineate physical intervals [37]. All annotated genes within these genomic regions were subjected to reciprocal best BLAST analysis against the *A. thaliana* genome to identify orthologues. Functional prioritization was conducted by cross-referencing these orthologues with experimentally validated GSL-related genes in *Arabidopsis*, including biosynthetic enzymes (e.g. GSL-OH, AOP1/2/3, CYP79s), transporters, and transcriptional regulators. Synteny analysis with reported GSL gene clusters in *Brassica* species (*B. rapa* and *B. napus*) further refined candidate selection. Genes recurrently mapped across overlapping QTLs were prioritized as core candidates underlying GSL variation.

### Candidate genes amplification, variation analysis, and phylogenetic tree construction

Candidate genes were amplified using specific primers (Supplementary Table S6) designed with Oligo 7 software [38], followed by PCR amplification under optimized conditions (98°C for 3 min; 35 cycles of 98°C/10 s, 53°C/20 s, 72°C/90 s). Following agarose gel electrophoresis, target PCR amplicons were excised and purified using the E.Z.N.A. ®Gel Extraction Kit D2500-01 (Omega Bio-Tek, Norcross, GA, USA), followed by ligation into TA-cloning vectors with the TA Cloning Kit C601-02 (Vazyme, Nanjing, China). Transformed competent cells were cultured overnight, and plasmid-containing bacterial suspensions were submitted to Tsingke Biotechnology Co., Ltd (Beijing, China) for bidirectional Sanger sequencing on an ABI 3730xl platform. Genetic variations were identified by aligning reads to HDEM reference genome, and functionally annotated with SnpEff (v5.1) to predict missense or frameshift mutations [39]. The coding sequences of three *AOP*-related candidate genes (*BolC9t53177H*, *BolC9t53178H*, and *BolC9t53179H*) from three parental lines (B58-6, PC14, J1402) and their *Arabidopsis* homologs were aligned using the MUSCLE algorithm in MEGA-X software [40]. The maximum likelihood phylogenetic tree was constructed with 1000 bootstrap replicates to confirm the evolutionary relationships.

### KASP markers development for the GRA, GNA, and PRO glucosinolates in *B. oleracea*

To establish robust molecular markers for GRA, GNA, and PRO in *B. oleracea*, we leveraged our previously published genome-wide resequencing data [41] to identify SNPs within candidate genes. KASP primers were designed using the LGC platform (IntelliQube, LGC Biosearch Technologies, Hoddesdon, UK), adhering to published protocols for primer optimization and genotyping [24]. SNP selection followed stringent criteria: (i) target loci were required to lack adjacent polymorphisms within a 50-bp flanking window to minimize off-target amplification and (ii) primer pairs were engineered to maintain a minimum GC content of 30% for balanced annealing efficiency. The resulting KASP markers were deployed to genotype a diverse panel of 104 *B. olearcea* accessions. Marker reliability was rigorously evaluated by correlating genotypic data with untargeted GSL profiling data (unpublished data), employing *T* tests to quantify associations between allelic variants and metabolite accumulations.

## Supplementary Material

Web_Material_uhaf232

## Data Availability

The data used to support the findings of this study are included within the article.
