# Supplementary material for: QTL and candidate gene analysis unveil genetic control of floret aliphatic glucosinolate side-chain modification in Brassica oleracea through multiparent F₂ populations
Source: Hortic Res. 2025 Sep 3;12(12):uhaf232. doi: 10.1093/hr/uhaf232 (PMC12682067; doi:10.1093/hr/uhaf232)
Supplement: Web_Material_uhaf232 [file web_material_uhaf232.zip › R2_Supplemental Figures.pdf]

Supplemental Fig. S1 Phenotypic distribution of the ratios for GRA, GNA, and PRO contents in JB-F_2 and GJ-F_2 populations

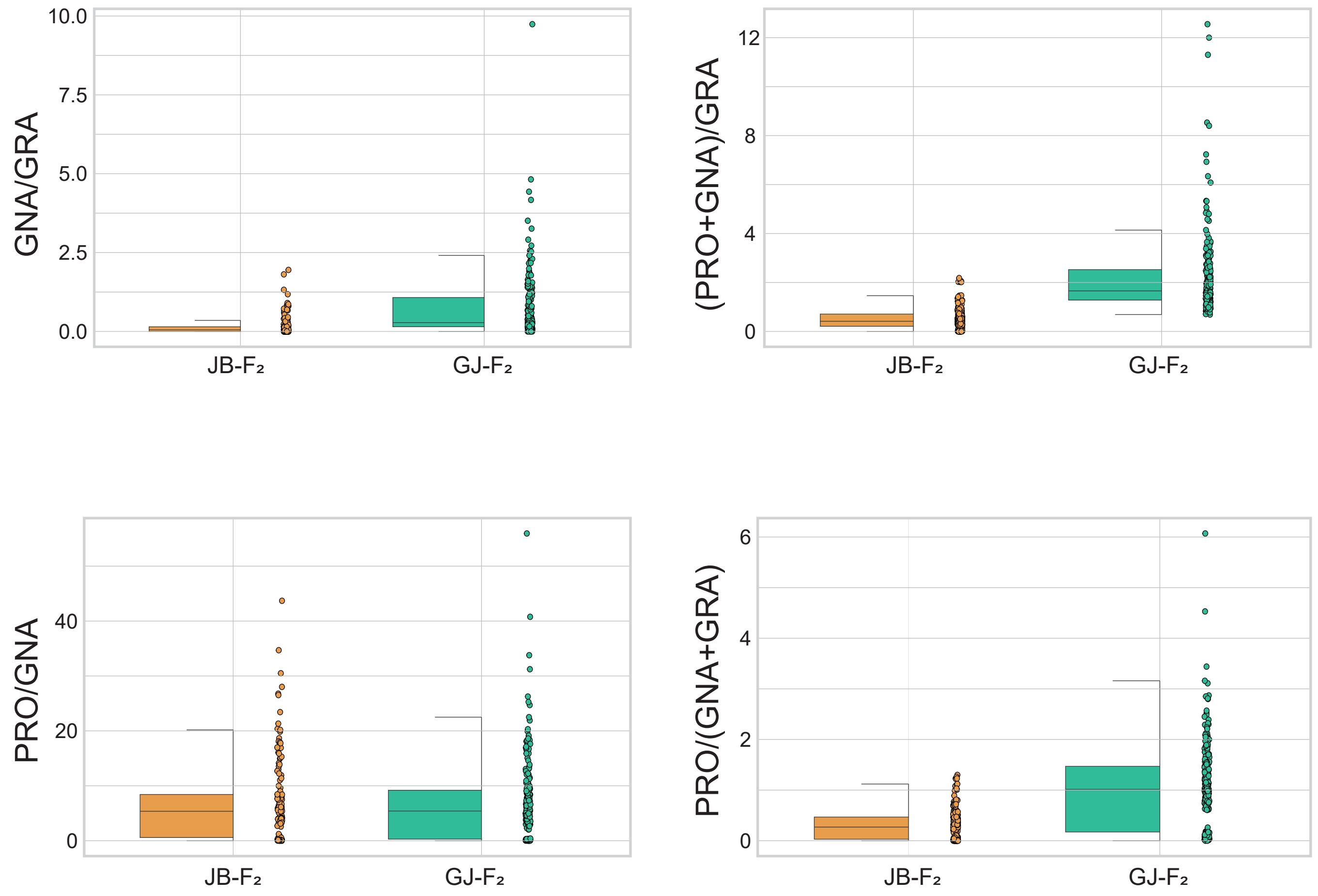

Supplemental Fig. S2 The results of aligning the amino acid sequence of the candidate genes in the HDEM reference genome to *Arabidopsis thaliana*

a

```
Query= BolC9t53177H
Length=440                                                                    Score E
Sequences producing significant alignments:                                 (Bits) Value
AT4G03060.1 | Symbols: AOP2 | 2-oxoglutarate (2OG) and Fe(II)-dep... 468 2e-164
AT4G03050.2 | Symbols: AOP3 | 2-oxoglutarate (2OG) and Fe(II)-dep... 387 7e-133
AT4G03050.1 | Symbols: AOP3 | 2-oxoglutarate (2OG) and Fe(II)-dep... 286 2e-94
AT4G03070.1 | Symbols: AOP1, AOP, AOP1.1 | 2-oxoglutarate (2OG) a... 230 3e-72
AT1G52820.1 | Symbols: | 2-oxoglutarate (2OG) and Fe(II)-dependen... 191 2e-57
AT1G52810.1 | Symbols: | 2-oxoglutarate (2OG) and Fe(II)-dependen... 151 2e-42
```

The result of aligning the amino acid sequence of the *BolC9t53177H* gene in the HDEM reference genome to *Arabidopsis thaliana*

b

```
Query= BolC9t53178H
Length=322
                                                                      Score E
Sequences producing significant alignments:                         (Bits) Value
AT4G03070.1 | Symbols: AOP1, AOP, AOP1.1 | 2-oxoglutarate (2OG) a... 503 0.0
AT1G52820.1 | Symbols: | 2-oxoglutarate (2OG) and Fe(II)-dependen... 384 2e-134
AT1G28030.1 | Symbols: | 2-oxoglutarate (2OG) and Fe(II)-dependen... 280 2e-93
AT1G52810.1 | Symbols: | 2-oxoglutarate (2OG) and Fe(II)-dependen... 256 2e-84
AT1G52790.1 | Symbols: | 2-oxoglutarate (2OG) and Fe(II)-dependen... 254 3e-83
AT1G52800.1 | Symbols: | 2-oxoglutarate (2OG) and Fe(II)-dependen... 249 3e-81
```

The result of aligning the amino acid sequence of the *BolC9t53178H* gene in the HDEM reference genome to *Arabidopsis thaliana*

c

```
  Query= BolC9t53179H
Length=322
                                                                       Score E
Sequences producing significant alignments:                         (Bits) Value
AT4G03070.1 | Symbols: AOP1, AOP, AOP1.1 | 2-oxoglutarate (2OG) a... 511 0.0
AT1G52820.1 | Symbols: | 2-oxoglutarate (2OG) and Fe(II)-dependen... 401 5e-141
AT1G28030.1 | Symbols: | 2-oxoglutarate (2OG) and Fe(II)-dependen... 285 3e-95
AT1G52800.1 | Symbols: | 2-oxoglutarate (2OG) and Fe(II)-dependen... 255 1e-83
AT1G52810.1 | Symbols: | 2-oxoglutarate (2OG) and Fe(II)-dependen... 251 2e-82
AT1G52790.1 | Symbols: | 2-oxoglutarate (2OG) and Fe(II)-dependen... 249 1e-81
```

The result of aligning the amino acid sequence of the *BolC9t53179H* gene in the HDEM reference genome to *Arabidopsis thaliana*

d

```
Query= BolC3t13531H
Length=375
                                                                       Score E
Sequences producing significant alignments:                         (Bits) Value
AT2G25450.1 | Symbols: GSL-OH | glucosinolate hydroxylase | chr2:... 557 0.0
AT2G30830.1 | Symbols: | 2-oxoglutarate (2OG) and Fe(II)-dependen... 526 0.0
AT2G30840.1 | Symbols: | 2-oxoglutarate (2OG) and Fe(II)-dependen... 504 3e-180
AT1G06620.1 | Symbols: | 2-oxoglutarate (2OG) and Fe(II)-dependen... 449 1e-158
AT1G06650.2 | Symbols: | 2-oxoglutarate (2OG) and Fe(II)-dependen... 432 9e-152
AT1G06645.1 | Symbols: | 2-oxoglutarate (2OG) and Fe(II)-dependen... 428 4e-150
```

The result of aligning the amino acid sequence of the *BolC3t13531H* gene in the HDEM reference genome to *Arabidopsis thaliana*

Supplemental Fig. S3 Variation analysis after aligning the amino acid sequences in genes *BolC9t53178H* and *BolC9t53179H* to the HDEM reference genome

BolC9t53178H-HDEM-Pro	MDSDSLLPLSESLELPVIDFSDQNLAPGTSKWDEVKDDVRKALEDYGCFQAYVDKVSNIELNKPVFEAME	70
BolC9t53178H-B58-6-Pro	MDSDSLLPLSESLELPVIDFSDQNLAPGTSKWDEVKDDVRKALEDYGCFQAYVDKVSNIELNKPVFEAME	70
BolC9t53178H-J1402-Pro	MDSDSLLPLSESLELPVIDFSDQNLAPGTSKWDEVKDDVRKALEDYGCFQAYVDKVSNIELNKPVFEAME	70
BolC9t53178H-PC14-Pro	MDSDSLLPLSESLELPVIDFSDQNLAPGTSKWDEVKDDVRKALEDYGCFQAYVDKVSNIELNKPVFEAME	70
BolC9t53178H-HDEM-Pro	ELFDLPVQTKQRNVSSKPLHGYLSHNLYQSLGIEEANDAEKVNYFTQQLWPDHGNKSISETMHKFSERSV	140
BolC9t53178H-B58-6-Pro	ELFDLPVQTKQRNVSFKPLHGYLSHNLYQSLGIEEANDAEKVNYFTQQLWPDHGNKSISETMHKFSERSV	140
BolC9t53178H-J1402-Pro	ELFDLPVQTKQRNVSSKPLHGYLSHNLYQSLGIEEANDAEKVNYFTQQLWPDHGNKSISETMHKFSERSV	140
BolC9t53178H-PC14-Pro	ELFDLPVQTKQRNVSSKPLHGYLSHNLYQSLGIEEANDAEKVNYFTQQLWPDHGNKSISETMHKFSERSV	140
BolC9t53178H-HDEM-Pro	ELDVMARRMIMESFGIEKYLDEHLNSTYYVLRLMKYTSAPDDDVEETKLGLLSHTDKSITTILHQYEVDG	210
BolC9t53178H-B58-6-Pro	ELDVMARRMMMESFGIEKYLDEHLNSTYYVLRLMKYTSAPDDDVEETKLGLLSHTDKSITTILHQYEVDG	210
BolC9t53178H-J1402-Pro	ELDVMARRMIMESFGIEKYLDEHLNSTYYVLRLMKYTSAPDDDVEETKLGLLSHTDKSITTILHQYEVDG	210
BolC9t53178H-PC14-Pro	ELDVMARRMIMESFGIEKYLDEHLNSTYYVLRLMKYTSAPDDDVEETKLGLLSHTDKSITTILHQYEVDG	210
BolC9t53178H-HDEM-Pro	LEIKTKDEKWIKVKPSQHCFIIMVGDFLCALLNGRLHSPRHRVLMTAKKTRYSTAMFSVPKQGVIIDSPE	280
BolC9t53178H-B58-6-Pro	LEIKTKDEKWIKVKPSQHCFIIMVGDFLCALLNGRLHSPRHRVLMTAKKTRYSTAMFSVPKQGVIIDSPE	280
BolC9t53178H-J1402-Pro	LEIKTKDEKWIKVKPSQHCFIIMVGDFLCALLNGRLHSPRHRVLMTAKKTRYSTAMFSVPKQGVIIDSPE	280
BolC9t53178H-PC14-Pro	LEIKTKDEKWIKVKPSQHCFIIMVGDFLCALLNGRLHSPRHRVLMTAKKTRYSTAMFSVPKQGVIIDSPE	280
BolC9t53178H-HDEM-Pro	ELVDEEHPRMFKPFEYNEFINFFHSEAGRKAESALHAFCAL	321
BolC9t53178H-B58-6-Pro	ELVDEEHPRMFKPFEYNEFINFFHSEAGRKAESALHAFCAL	321
BolC9t53178H-J1402-Pro	ELVDEEHPRMFKPFEYNEFINFFHSEAGRKAESALHAFCAL	321
BolC9t53178H-PC14-Pro	ELVDEEHPRMFKPFEYNEFINFFHSEAGRKAESALHAFCAL	321

BolC9t53179H-HDEM-Pro	MDSDSLLPLSESLQLPVIDFSDQNLTPGTSKWDKVKADVRKALEDYGCFQAYVDKVSNIELDKSVYEAME	70
BolC9t53179H-B58-6-Pro	MDSDSLLPLSESLQLPVIDFSDQNLTPGTSKWDKVKADVRKALEDYGCFQAYVDKVSNIELDKSVYEAME	70
BolC9t53179H-J1402-Pro	MDTDSLLPFSESLQLPVIDFSDQNLTPGTSKWDKVKADVRKALEDYGCFQAYVDKVSNIELDKSVYEAME	70
BolC9t53179H-PC14-Pro	MDSDSLLPLSESLQLPVIDFSDQNLTPGTSKWDKVKADVRKALEDYGCFQAYVDKVSNIELDKSVYEAME	70
BolC9t53179H-HDEM-Pro	KLFDLPVQTKQRNVSSKPFHGYLSHNLYQSLGIEDANVAEKVNDFIQLLWPDHGNKSISEMMHKFSTQLV	140
BolC9t53179H-B58-6-Pro	KLFDLPVQTKQRNVSSKPFHGYLSHNLYQSLGIEDANVAEKVNDFIQLLWPDHGNKSISEMMHKFSTQLV	140
BolC9t53179H-J1402-Pro	KLFDLPVQTKQRNVSSKPFHGYLSHNLYQSLGIEDANVAEKVNDFIQLLWPDHGNKSISEMMYKFSEQLV	140
BolC9t53179H-PC14-Pro	KLFDLPVQTKQRNVSSKPFHGYLSHNLYQSLGIEDANVAEKVNDFIQLLWPDHGNKSISEMMHKFSTQLV	140
BolC9t53179H-HDEM-Pro	ELDVMVRRMIMESFGVEKYLDEHLNSTNYLFRMMKYTAPPDDDVEEAKLGLRSHTDKNIITILHQYEVDG	210
BolC9t53179H-B58-6-Pro	ELDVMVRRMIMESFGLEKYLEEHLNSTNYLFRMMKYTAPPDDDVEEAKLGLRSHTDKNIITILHQYEVDG	210
BolC9t53179H-J1402-Pro	ELDVMVRRMIMESFGVEKYLDEHLNSTNYLFRMMKYTAPPDDDVEEAKLGLRSHTDKNIITILHQYEVDG	210
BolC9t53179H-PC14-Pro	ELDVMVRRMIMESFGVEKYLDEHLNSTNYLFRMMKYTAPPDDDVEEAKLGLRSHTDKNIITILHQYEVDG	210

BolC9t53179H-HDEM-Pro	LEIMTKDAKWIKVKPSQHSFIIMVGDSLCALLNGRLYSPYHRVLMVAKKTRYSTAMFSVPKSGAIIDSPE	280
BolC9t53179H-B58-6-Pro	LEIMTKDCQWIKVKPSQHSFIIMVGDSLCALLNGRLYSPYHRVLMVAKKTRYSTAMFSVPKSGAIIDSPE	280
BolC9t53179H-J1402-Pro	LEIMTKDCQWIKVKPSQHSFIIMVGDSLCALLNGRLYSPYHRVLMVAKKTRYSTAMFSVPKSGVIIDSPE	280
BolC9t53179H-PC14-Pro	LEIMTKDAKWIKVKPSQHSFIIMVGDSLCALLNGRLYSPYHRVLMVAKKTRYSTAMFSVPKSGAIIDSPE	280

BolC9t53179H-HDEM-Pro	EVVDEEHPRMFKPFEYMDFLNFFHSEAGRRVESTLHAFCAL	321
BolC9t53179H-B58-6-Pro	EVVDEEHPRMFKPFEYMDFLDFFHSEAGRRVESTLHAFCAL	321
BolC9t53179H-J1402-Pro	EVVDEEHPRMFKPFEYMDFLNFFHSEAGSRVESTLHAFCAL	321
BolC9t53179H-PC14-Pro	EVVDEEHPRMFKPFEYMDFLNFFHSEAGRRVESTLHAFCAL	321
